# T‐cells infiltration mediates the association between neutrophil/lymphocyte ratio and survival in gastric cancer

**DOI:** 10.1002/cam4.6228

**Published:** 2023-06-12

**Authors:** Qifei He, Longtao Huangfu, Biao Fan, Qianzheng Zhuang, Liu He, Lin Li, Wei You, Xiaofang Xing

**Affiliations:** ^1^ Key Laboratory of Carcinogenesis and Translational Research (Ministry of Education), Gastrointestinal Cancer Center Peking University Cancer Hospital and Institute Beijing China; ^2^ Department of Bone Joint and Musculoskeletal Tumor The First Affiliated Hospital of Shenzhen University, Shenzhen Second People's Hospital Shenzhen China; ^3^ Department of Gastroenterology, Aerospace Center Hospital Peking University Aerospace School of Clinical Medicine Beijing China

**Keywords:** gastric cancer, immune infiltrates, neutrophil/lymphocyte ratio, prognosis

## Abstract

**Background:**

Neutrophil/lymphocyte ratio (NLR) is a vital index for systemic inflammation and a prognostic indicator for gastric cancer (GC). Despite the abundant literature on NLR's prognostic value for GC, the underlying factors mediating its impact on survival remain unclear. The objective of this study was to analyze the role of NLR in different prognostic models and subgroups, and investigate the mediating effects of immune infiltrates between NLR and survival.

**Methods:**

A total of 924 patients who underwent D2 lymph node resection were enrolled in this study. According to the level of NLR, patients were divided into two groups, the high and low NLR groups. Clinical parameters, indexes related to immune infiltrates, and survival were compared between the two groups. Prognostic models, interaction analysis, and mediating effects analysis were performed to investigate the clinical association of NLR, immune infiltrates, and survival.

**Results:**

The infiltration of CD3+ and CD8+ T cells was significantly different in the two NLR groups. The level of NLR was an independent prognostic predictor of GC. In addition, an interaction effect exists between NLR and MMR status on the prognosis of GC (*p*‐interaction <0.01). Lastly, the mediating effect analysis revealed that the infiltration level of CD3+ T cells was the mediating factor between NLR and survival (*p* < 0.001).

**Conclusions:**

The level of NLR is an independent prognostic predictor of GC. The effect of NLR on prognosis is partly mediated by CD3+ T‐cell infiltration.

## Graphical abstract

The level of NLR is an independent prognostic predictor of gastric cancer. The effect of NLR on patient prognosis is partly through influencing CD3+ T‐cells infiltration.

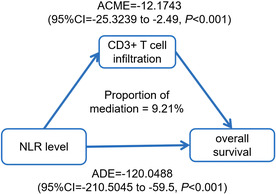

## 1 BACKGROUND

Gastric cancer (GC) poses a significant global health concern, with over one million new cases diagnosed annually.^1^ To guide postoperative treatment and predict patients' prognosis, multiple staging, and prognostic systems have been established.

The TNM staging system is one of the most commonly used prognostic and staging systems in GC. Meanwhile, numerous novel prognostic factors, such as neutrophil‐to‐lymphocyte ratio (NLR), have emerged.^2^ NLR is one of the systemic inflammation markers that correlate with clinical outcomes of various cancers, including gastric cancer, colorectal cancer, pancreatic cancer, and liver cancer.^3^, ^4^, ^5^ The prognostic role of NLR in GC has been extensively reported by different groups.^6^, ^7^, ^8^, ^9^ However, the underlying mechanisms by which NLR in GC impacts prognosis remain uncertain. Other indexes, such as tumor‐infiltrating lymphocytes (TIL) and markers of systemic inflammation, have been recognized as prognostic factors as well.^10^, ^11^ The tumor immune microenvironment plays a pivotal role in tumor initiation and progression. Studies indicated that markers of microenvironment function as independent prognostic predictors for patient survival across multiple malignancies.^12^, ^13^, ^14^

As a multitude of inflammatory cells are recognized to migrate from peripheral blood to local tissues through systemic circulation, NLR has been observed to correlate with the density of CD4+ immune cells or the presence of tertiary lymphoid structures (TLS).^15^, ^16^ This suggests the underlying association between TIL and NLR in the prognosis of gastric cancer. However, evidence is still lacking to elucidate the extent of TIL's impact on the prognosis of NLR among GC patients. In addition, beyond TLS, immune checkpoint inhibitors are another critical index for identifying patients who are most likely to benefit from cancer immunotherapy.^17^ Further investigations are required to evaluate the relationship between NLR and immune infiltrates or immune checkpoints, and their respective roles in the prognosis of gastric cancer.

Therefore, in the current study, our aim is to validate the prognostic significance of NLR, assess the association between NLR and TIL, and examine the mediating effect of lymphocytic infiltrates on the relationship between NLR and survival in gastric cancer. Additionally, we intend to investigate the prognostic impact of NLR on various subgroups categorized by immune checkpoints and pathological features.

## 2 METHODS

### 2.1 Patient selection

We conducted a retrospective study in eligible GC patients who underwent gastrectomy at Peking University Cancer Hospital between June 2003 and December 2012. The patients were selected from Xiaofang Xing's previous research studying T‐cell infiltration (TCI) in GC.^18^ The inclusion criteria of the patient include patients with (i) available FFPE tissues; (ii) histologic identification of the adenocarcinoma; (iii) without preoperative chemotherapy or radiotherapy. The exclusion criteria were the patients (a) without blood index information, such as neutrophil counts and lymphocyte counts, within 2 weeks before the operation; (b) with preoperative sepsis, confirmed systemic infection, inflammatory conditions, and other types of cancer. In this study, all the cases were originally thought to be potentially resectable from preoperative radiography and received gastrectomy with D2 lymph node resection. Adjuvant therapy was typically administered in patients with locally advanced gastric cancer (postoperative pTNM stage II and stageIII). For those with stage I, adjuvant therapy was not performed after D2 radical gastrectomy. Some patients after surgery were found metastases intraoperatively or postoperatively, such as radiographically occult peritoneal metastases and positive peritoneal cytology, which were staged as pTNM stage IV (R1‐2) in our study.

The PUCH Ethics Committee approved the study (permission number 2019KT111), and all participants included in the present study have provided informed consent. Follow‐up data were retrieved from hospital records.

### 2.2 Definition of neutrophil‐to‐lymphocyte ratio

**FIGURE 1:**
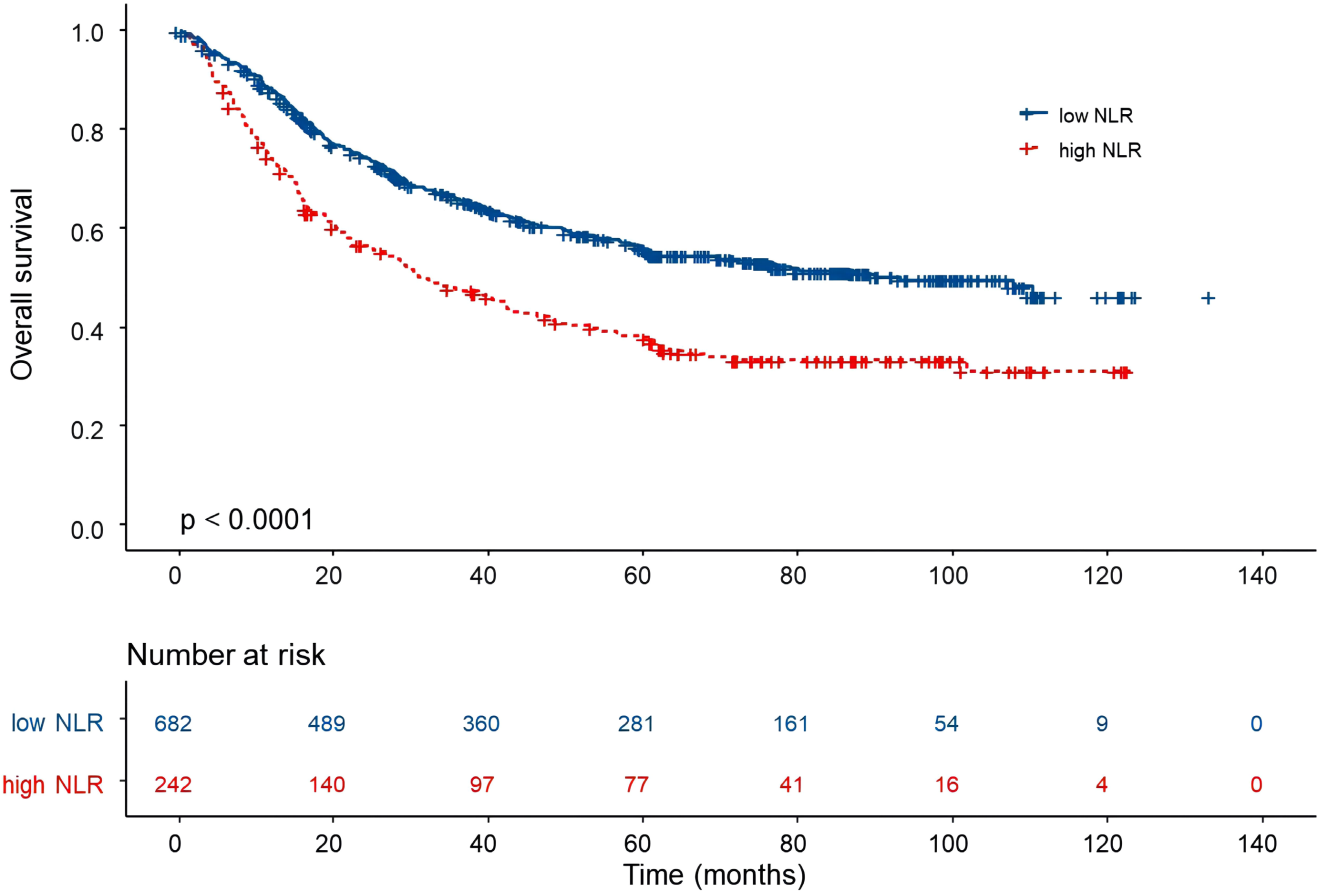
Kaplan–Meier curves of different NLR levels.

We used the neutrophil‐to‐lymphocyte ratio (NLR) as the marker of systemic inflammation in this study, which was calculated as the neutrophil count ratio to the lymphocyte count. An optimal cutoff value was defined to classify the samples into two groups (high NLR vs. low NLR) for the value of NLR using X‐tile plots based on the association with the patients' overall survival. The X‐tile plots can automatically generate the optimum cutoff point for continuous variables according to the highest χ^2^ value (minimum *p* value) defined by Kaplan–Meier survival analysis and log‐rank test. The cutoff value for the NLR was determined to be 3.09 in our study, with the low NLR (<3.09) group showing a significantly higher survival rate than that with high NLR (>3.09) in Kaplan–Meier analysis (*p* < 0.001, Figure 1).

### 2.3 Immunohistochemical staining

We built tissue microarrays (TMAs) from surgical specimens preserved in paraffin blocks. Then, we performed immunohistochemistry on the section of TMAs after deparaffinization. Arrays were stained with the following primary antibodies: antiCD3 antibody (NCL‐CD3‐565, Leica), anti‐CD8 antibody (CRM311C, Biocare Medical), anti‐MutL homolog 1 (MLH1), antibody (IR079, DAKO), anti‐mutS homolog 2 (MSH2) antibody (IR085, DAKO), anti‐mutS homolog 6 (MSH6) antibody (IR086, DAKO), and anti‐PD‐L1 antibody (SP142, Roche). Details of the protocols and scoring schema for the hMLH1, MSH2, MSH6, PDL1, CD3, and CD8 were presented in the previous study of Xiaofang Xing, et al.^18^

### 2.4 Evaluation of immunohistochemistry staining

The densities of CD3+ and CD8+ immune cell infiltration were assessed with the percentage immunoreactivity (positive cells/(positive cells + negative cells)*100), which was used as a surrogate for the extent of immune cell infiltration. A previously described image analysis system was used to identify all strained cores of immune cells and was performed as follows. Stained TMAs slides were scanned at ×20 magnification with an Aperio XT digital slide scanner and subjected to automatic image analysis to identify and quantify immunoreactivity. Brown (immunopositive) pixels, blue (immunonegative) pixels, and white (empty space) pixels in slides were discriminated against by the TMAs system, an in‐house developed software. A senior GI pathologist reviewed all cores after the image analysis process and confirmed that (i) the detection of the brown staining had been performed accurately by the software and (ii) to exclude all cores which contained tumor cells.

The expression of MMR genes was briefly defined as follows. MMR‐proficient (pMMR) status referred to simultaneously expressing MLH1, MSH2, and MSH6, while MMR‐deficient (dMMR) states were defined as the loss of MLH1 and loss of both MSH6 and MSH2 expression. The immunostaining of PD‐L1 in tumors was rated as presence or absence. The consensus among three pathologists determined the final interpretation. A high level of consistency and few discrepant cases (<5%) was reached after a joint review.

### 2.5 Statistical analysis

We computed the Kaplan–Meier curves and log‐rank test based on X‐tile software to compare OS between low and high NLR groups. Distributions of continuous variables between two groups were described as mean or median, and standard deviation or interquartile range and compared using the *t*‐test or, in cases of non‐normality, the Mann–Whitney test. Categorical variables were described as numbers with percentages and compared using the χ^2^ test or Fisher's exact test. We performed univariate and multivariate Cox proportional hazard regression models to calculate the hazard ratios (HR). In the Cox regression models, we considered the covariables associated with NLR level, survival outcome, and tumor microenvironment, including age (<65 vs. > = 65), gender (female vs. male), pTNM stage (I vs. II vs. III vs. IV), site (EGJ vs. GC), Lauren type (diffuse vs. Intestinal vs. mixed), differentiation grade (poorly or not), MMR status (proficient vs. Deficient), PDL1 expression (positive vs. negative), and the densities of CD3+ and CD8+ immune cells. Moreover, we used mediation analysis to explore the role of TCI that explained the NLR disparities in the survival rate among GC patients. We further performed subgroup analyses to investigate the HR of different NLR levels across different subgroups stratified by age, gender, tumor site, pTNM stage, differentiation grade, Lauren type, MMR status, and PDL1 expression. We also conducted an interaction test to evaluate the heterogeneity of treatment effects across the subgroups.

We performed the X‐tile plots with the X‐tile software version 3.6.1 (Yale University School of Medicine, New Haven, CT, USA). We conducted all the other statistical tests with R software version 4.0.1 (with “mediation” package for mediation analysis, “survivalROC” package and “timeROC” package for ROC curve, “survival” package for Cox regression, and “ggplot2” and “forestplot” for plot figures) (R Foundation for Statistical Computing, Vienna, Austria). Statistical significance was declaimed with two‐sided *p* < 0.05 for all tests.

## 3 RESULTS

### 3.1 Patient characteristics

**FIGURE 2:**
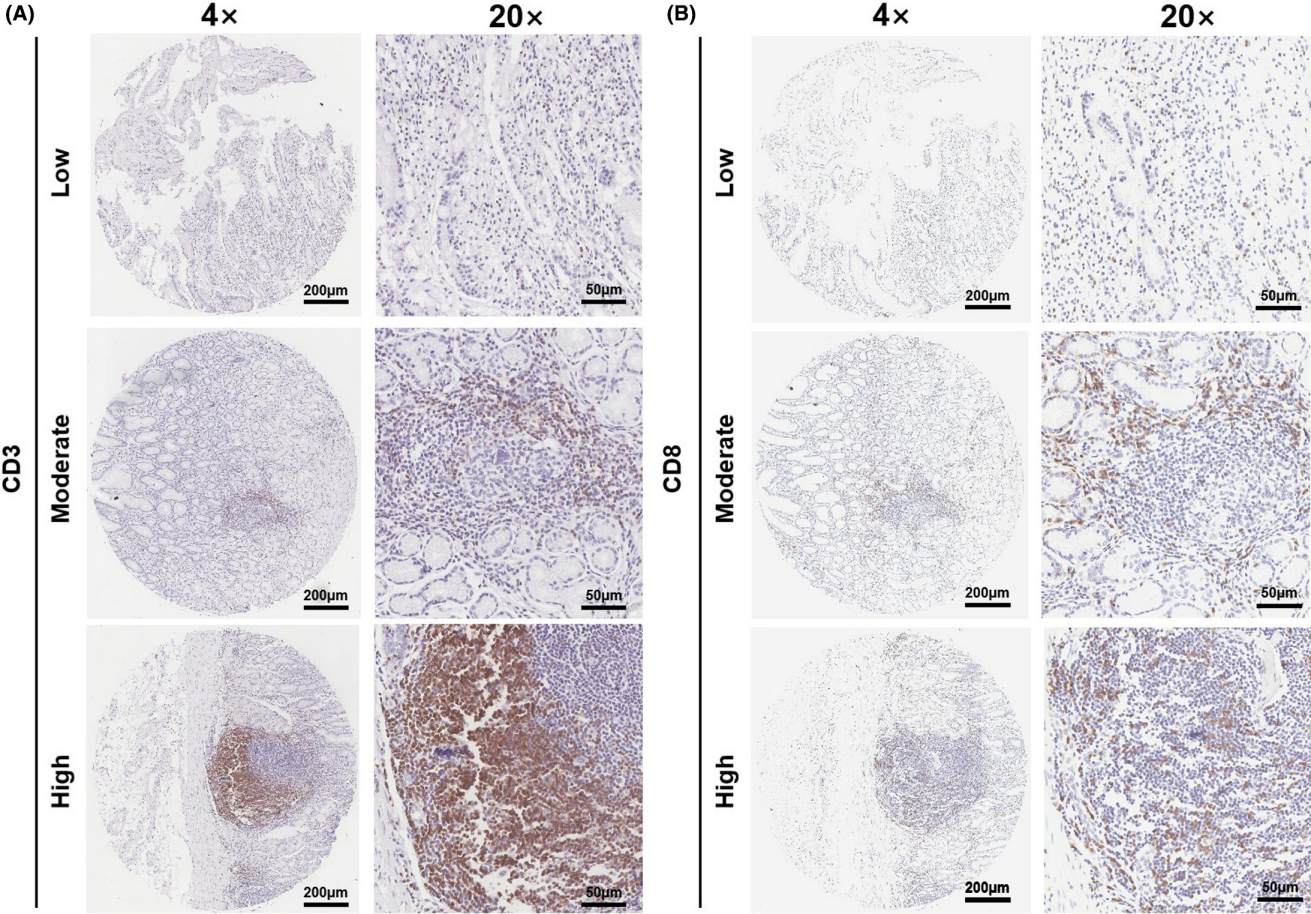
CD3 and CD8 expression in gastric carcinoma by immunohistochemistry (×4 and ×20). (A) The representative images of CD3+ T cells with different staining intensities; (B) The representative images of CD8 + T lymphocytes with different staining intensities.

A total of 924 patients with resected FFPE tissue and preoperative blood indexes were included in our study. Table 1 summarizes the patient‐related, tumor‐related, and microenvironment‐related characteristics. The mean age was 60.16 years (range 22–89 years). Among all patients, female patients accounted for 28.0%, and those aged 65 years or older made up 36.9%. Most tumors were located at the gastric part (76.3%) and were identified as an intestinal type (55.3%) or diffuse type (24.7%) by the Lauren classification. Approximately half of the patients (46.4%) exhibited poor differentiation. MMR‐deficient tumor and PDL1‐positive tumor were observed in 91 patients (9.8%) and 351 patients (38.0%), respectively. The percentages of cancers by stage were as follows: 11.8% for stage I cancer, 27.8% for stage II cancer, 52.8% for stage III cancer, and 7.6% for stage IV cancer. The mean density of CD3+ and CD8+ T cells were 12.79% and 11.03%, respectively (Figure 2).

**TABLE 1:** Clinicopathological and molecular features according to NLR level.

Variable	N	Overall, *N* = 924^a^	Low NLR, *N* = 682^a^	High LNR, *N* = 242^a^	*p* value^b^
Age	924				<0.001
<65		583 (63.1%)	453 (66.4%)	130 (53.7%)	
> = 65		341 (36.9%)	229 (33.6%)	112 (46.3%)	
Gender	924				0.637
Female		259 (28.0%)	194 (28.4%)	65 (26.9%)	
Male		665 (72.0%)	488 (71.6%)	177 (73.1%)	
pTNM	924				0.004
I		109 (11.8%)	87 (12.8%)	22 (9.1%)	
II		257 (27.8%)	198 (29.0%)	59 (24.4%)	
III		488 (52.8%)	357 (52.3%)	131 (54.1%)	
IV		70 (7.6%)	40 (5.9%)	30 (12.4%)	
Site	924				0.100
EGJ		219 (23.7%)	171 (25.1%)	48 (19.8%)	
GC		705 (76.3%)	511 (74.9%)	194 (80.2%)	
Lauren type	924				0.379
Diffuse		228 (24.7%)	170 (24.9%)	58 (24.0%)	
Intestinal		511 (55.3%)	367 (53.8%)	144 (59.5%)	
Mixed		168 (18.2%)	131 (19.2%)	37 (15.3%)	
(Missing)		17 (1.8%)	14 (2.1%)	3 (1.2%)	
Differentiation	924				0.572
Poorly		429 (46.4%)	313 (45.9%)	116 (47.9%)	
Moderately or well		450 (48.7%)	338 (49.6%)	112 (46.3%)	
(Missing)		45 (4.9%)	31 (4.5%)	14 (5.8%)	
MMR	924				0.642
Proficient		706 (76.4%)	518 (76.0%)	188 (77.7%)	
Deficient		91 (9.8%)	66 (9.7%)	25 (10.3%)	
(Missing)		127 (13.7%)	98 (14.4%)	29 (12.0%)	
CD3	924	12.79% (9.02)	13.33% (9.21)	11.28% (8.30)	<0.001
CD8	924	11.03% (7.77)	11.48% (8.27)	9.75% (5.96)	0.022
PDL1	924				0.285
Negative		573 (62.0%)	416 (61.0%)	157 (64.9%)	
Positive		351 (38.0%)	266 (39.0%)	85 (35.1%)	

Abbreviations: EGJ, Esophagogastric junction; GC, gastric cancer; MMR, mismatch repair; NLR, neutrophil‐to‐lymphocyte ratio; PDL1, programmed cell death‐ligand 1; pTNM, pathological TNM stage.

^a^ Mean (SD); *n* (%).

^b^ Wilcoxon rank sum test; Pearson's chi‐squared test; Fisher's exact test.

Using the defined cutoff value of NLR (3.09), we identified 682 patients (73.8%) as having a low NLR and 242 patients (26.2%) as having a high NLR. The high NLR group displayed a higher proportion of patients with old age (*p* < 0.001) and late pTNM stage (*p* = 0.004), relative to the low NLR group. The high density of CD3+ (*p* < 0.001) and CD8+ (*p* = 0.022) immune cell infiltration was also observed in the low NLR group. The NLR was not correlated with gender, tumor site, Lauren type, histologic differentiation degree, MMR status, or PDL1 expression.

### 3.2 NLR and survival outcomes

In the univariable Cox regression analysis, high NLR, old age, and high pTNM stage have significantly increased the risk of mortality (Figure 1 and Table 2). Gastric cancer patients with a high density of TCI, Lauren intestinal type, and PDL1 expression in tumors showed better survival (Table 2). Multivariate regression analysis indicated NLR, age stratification, pTNM stage, Lauren type, CD3+ TCI, and PDL1 expression in a tumor significantly associated with OS after controlling for the major confounders (Table 2).

**TABLE 2:** Univariate and multivariate analysis of overall survival (OS) rates.

Characteristic	Univariable			Multivariable		
	HR	95% CI	*p* value	HR	95% CI	*p* value
NLR	1.74	1.43, 2.12	<0.001	1.58	1.29, 1.93	<0.001
Age						
<65	—	—		—	—	
> = 65	1.49	1.24, 1.80	<0.001	1.64	1.35, 2.00	<0.001
Gender						
Female	—	—		—	—	
Male	1.13	0.92, 1.40	0.244	1.09	0.88, 1.36	0.425
pTNM						
I	—	—		—	—	
II	3.59	1.91, 6.73	<0.001	3.34	1.78, 6.27	<0.001
III	9.13	5.00, 16.68	<0.001	8.48	4.62, 15.5	<0.001
IV	24.16	12.65, 46.14	<0.001	23.1	12.0, 44.4	<0.001
Site						
EGJ	—	—		—	—	
GC	0.99	0.79, 1.23	0.908	0.85	0.68, 1.06	0.139
Differentiation						
Poorly	—	—		—	—	
Moderately or well	0.84	0.69, 1.01	0.062	1.00	0.80, 1.25	0.999
(Missing)	0.7	0.43, 1.15	0.164	0.60	0.36, 1.00	0.051
Lauren type						
Diffuse	—	—		—	—	
Intestinal	0.75	0.6, 0.93	0.008	0.66	0.51, 0.85	0.002
Mixed	0.9	0.69, 1.18	0.449	0.87	0.65, 1.16	0.334
(Missing)	0.39	0.14, 1.05	0.062	0.62	0.22, 1.70	0.349
MMR						
Proficient	—	—		—	—	
Deficient	0.81	0.58, 1.13	0.206	0.85	0.60, 1.20	0.362
(Missing)	0.91	0.69, 1.21	0.527	0.80	0.60, 1.06	0.120
CD3	0.97	0.96, 0.98	<0.001	0.98	0.97, 0.99	0.004
CD8	0.97	0.96, 0.99	<0.001	1.00	0.99, 1.02	0.750
PDL1						
Negative	—	—		—	—	
Positive	0.69	0.57, 0.85	<0.001	0.78	0.64, 0.96	0.021

Abbreviations: CI, confidence interval; EGJ, Esophagogastric junction; GC, gastric cancer; HR, hazard ratio; MMR, mismatch repair; NLR, neutrophil‐to‐lymphocyte ratio; PDL1, programmed cell death‐ligand 1; pTNM, pathological TNM stage.

### 3.3 Subgroup analysis

**FIGURE 3:**
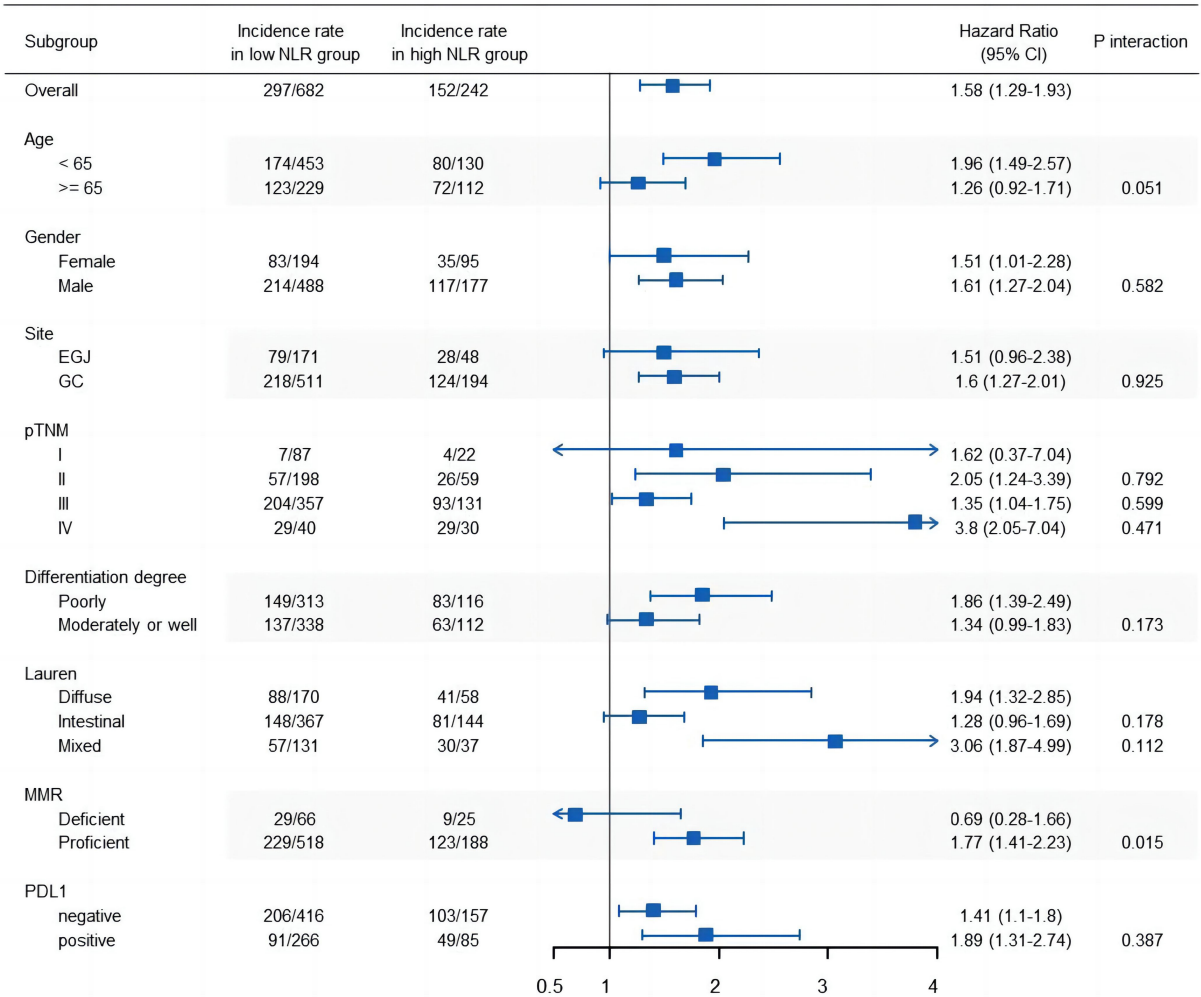
Forest plot of overall survival stratified by subgroups. Subgroup analyses and interaction analyses were conducted using patients' baseline characteristics.

We performed a subgroup analysis of the effect of NLR (high group vs. low group) according to age, gender, tumor site, pTNM stage, differentiation grade, Lauren type, MMR status, and PDL1 expression (Figure 3 and Figure S1). High NLR was associated with a worse prognosis across age, gender, tumor site, pTNM stage, differentiation grade, Lauren type, and PDL1 expression due to the values of HR greater than 1. In addition, subgroup analysis according to MMR status demonstrated high NLR improve OS among patients with dMMR (HR, 0.69; 95% CI, 0.28–1.66), but not in those with pMMR (HR, 1.77; 95% CI, 1.41–2.23), (*p*‐interaction <0.01), (Figure 3 and Figure S2).

### 3.4 Mediation analysis

**FIGURE 4:**
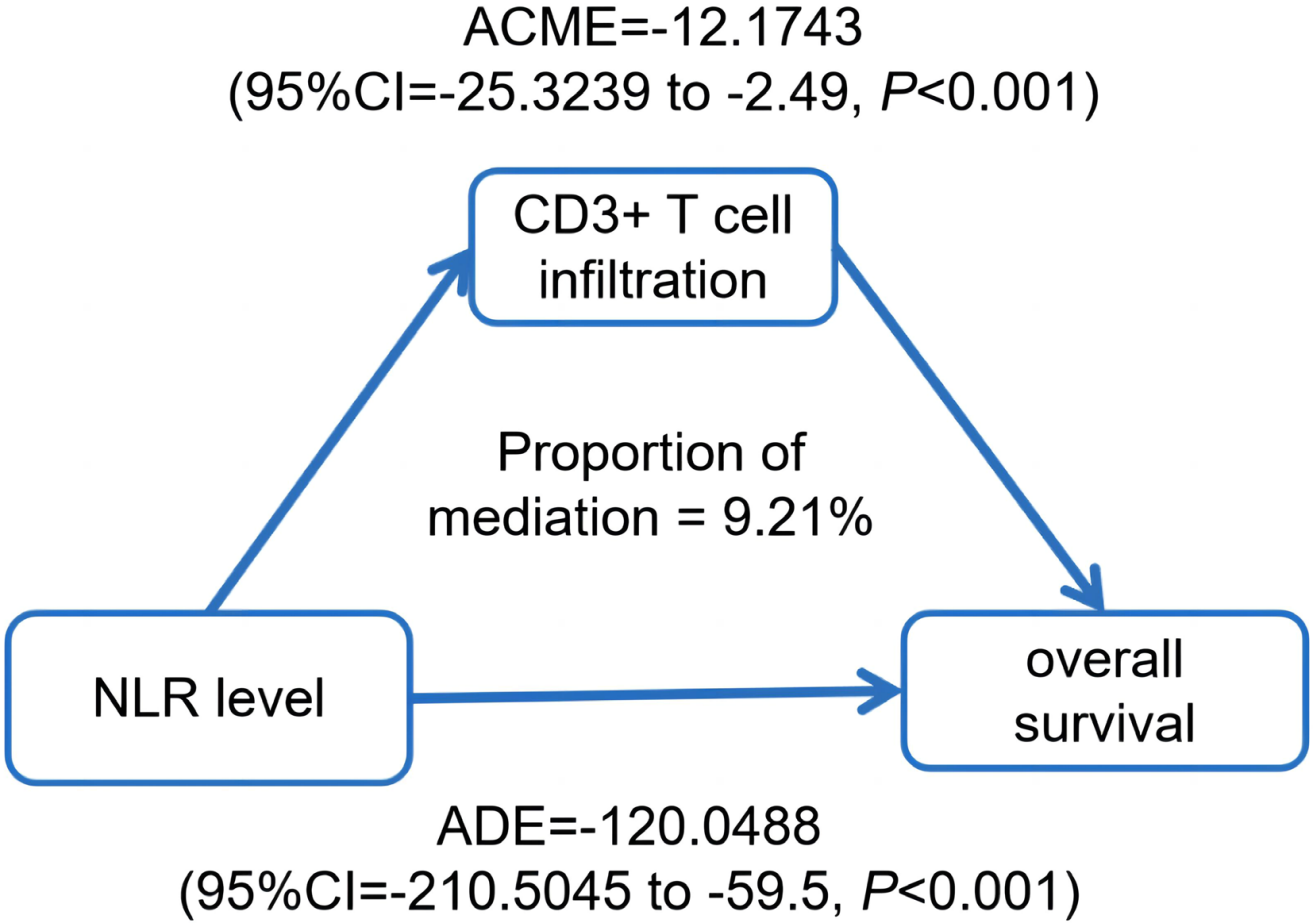
A causal mediation analysis of the CD3+ T‐cell infiltration between NLR and OS. ACME, average causal mediation effects; ADE, average direct effects.

According to the results of univariable and multivariable COX regression analyses, the mediator effect of TCI might exist in the relationship between NLR and OS, as following facts^1^: TCI was significantly correlated with the NLR (*p* < 0.05) and survival (*p* < 0.05), (Table 1 and Table 2)^2^; NLR was significantly associated with OS (*p* < 0.001) in univariable analysis (Table 2),^3^ the correlation intensity between NLR and OS weakened after controlling TCI in multivariable analysis (Table 2). Mediation analysis indicated that CD3+ TCI had significant mediation effects on the association between NLR and overall survival (*p* < 0.001), which explains about 9.21% of the association (Figure 4). CD3+ TCI is a mediator of the association between NLR and overall survival.

## 4 DISCUSSION

We revealed a higher NLR was associated with advanced‐stage gastric cancer. Infiltration of CD3+ and CD8+ T cells was significantly different in patients with different levels of NLR. Furthermore, through mediating effects analysis, we investigated the role of immune infiltrates in the prognosis value of NLR and statistically suggested that NLR affects tumor prognosis through immune infiltration statistically. These findings indicate that systemic inflammation involves in the development and progression of gastric cancer. The peripheral blood NLR could be used to reflect the cancer immune microenvironment and to predict the patient with a worse prognosis who might need aggressive postoperative adjuvant therapy.

In recent years, our understanding of the cancer immune microenvironment has greatly improved. Lymphoid regulatory cells including regulatory T, B, and NK cells in the tumor site have been reported to support cancer growth, migration, and metastasis.^19^ Cancer‐associated myeloid cells are involved in cancer cell biology, including proliferation, invasion, distant metastasis, and the development of resistance to therapy.^20^ STAT3 played a role in several cross talk levels between tumor cells and the immune microenvironment, and mediated tumor‐induced immunosuppression.^21^ Helicobacter pylori (HP) infection‐induced chronic inflammation is a significant factor in gastric cancer. The development of gastric cancer is attributed to genetic alterations caused by chronic inflammation, recruitment of immune cells, an imbalance between epithelial cell proliferation and apoptosis, and gastric colonization by enteric bacteria. Increased expression of pro‐inflammatory cytokines and chemokines such as IL‐17A, IL‐22, and IL‐1 family members IL‐1β is involved in gastric cancer progression.^22^ Thus, systemic inflammation might play an essential role in the gastric cancer microenvironment. Besides these pieces of molecular biology evidence, our results provided more information about systemic inflammation and tumor microenvironment from the perspective of clinical prognosis through mediation analysis.

Lymphopenia is an impaired cell‐mediated inflammatory response, whereas neutrophilia is an immune response that triggers tumorigenesis.^23^ NLR can be used to represent a balance between protumor and antitumor immunity. The NLR also can reflect the cancer burden and tumor biological behavior. Kei Nakamura, et al showed that NLR was associated with undifferentiated histology, advanced clinical T stage, and N stage.^24^ Kazuhiro Migita, et al concluded NLR might be associated with the extent of tumor spread at the time of recurrence.^25^ Consistent with previous studies, we found that a high level of NLR was associated with the late tumor stage. The TNM stage system is always used to predict the prognosis of cancer and guide postoperative treatment. Nevertheless, prognosis differs greatly among patients with the same tumor stage, which indicates that other prognostic factors should be included to optimize the system. Previous studies have reported the prognostic value of NLR in gastric cancer with the cutoff value set as 3 mg/dL.^15^, ^26^ Similarly, in our study, patients were also divided into two groups based on the level of NLR. NLR is an independent prognostic predictor of gastric cancer. Taken together, we conclude that NLR can be proposed and used as a predictor to stratify patients with different prognoses.

Lymphocytes are associated with a favorable prognosis in multiple tumors. The NLR is associated with the density of CD4+ T cells in gastric cancer.^15^ It also negatively correlated with CD8+ T cells infiltrating in biliary tract cancer.^27^ High NLR is significantly associated with high neutrophil infiltration and low CD3+ T cells in glioblastoma.^28^ Here, we showed that the infiltration of CD3+ and CD8+ T cells was both significantly different in patients with different levels of NLR. The infiltration level of CD3+ T cells was the mediating factor between NLR and survival prognosis. Thus, the NLR might serve as a valuable indicator for evaluating the immunoreactivity in the gastric cancer microenvironment.

The NLR was thought to be correlated with the tolerability and response to anticancer therapy in multiple cancers.^29^ NLR might be valuable for screening patients who will benefit from palliative chemotherapy. In our study, 88.2% of patients were with locally advanced/advanced gastric cancer. Many of them were treated with postoperative chemotherapy after D2 lymph node resection. Surprisingly, the effect of NLR on the patient prognosis prediction was affected by MMR status. MMR status is a mature biomarker for predicting the efficacy of immune checkpoint inhibitors.^30^ This might indicate that the NLR level is associated with the efficacy of immune therapy and that markers of systemic inflammation could be used to predict immune responses in cancer therapy.

There are some limitations in this study. Firstly, this is a retrospective study in which patient selection bias might exist. Secondly, the cutoff value of NLR is needed to be verified in a multicentric, independent cohort. Thirdly, we did not evaluate the postoperative dynamic changes in the NLR. Next, immunotherapy in gastric cancer is the research focuses currently. Although we found the association between NLR and immune infiltration in GC, the underlying mechanism, and the relationship between NLR and the efficacy of immunotherapy/chemotherapy are still obscure. More prospective studies of the neoadjuvant therapy for locally advanced GC and the immunochemotherapy for advanced GC should be designed to investigate the dynamic relationship among NLR, immune infiltration, and treatment. Lastly, the assessment of some reported systemic inflammation indicators, such as CRP, was not included in this study. Larger prospective studies are needed to evaluate these issues. However, despite the limitations mentioned above, we statistically evaluated the mediation effects of the tumor microenvironment on the association between systemic inflammation and overall survival for the first time. In addition, we described the relationship between NLR and immune checkpoints.

## 5 CONCLUSIONS

In summary, we revealed that the level of NLR is an independent prognostic predictor of gastric cancer. The effect of NLR on patient prognosis is partly through influencing CD3+ T‐cell infiltration.

## AUTHOR CONTRIBUTIONS

**Qifei He:** Data curation (equal); investigation (equal); validation (equal); writing – original draft (equal); writing – review and editing (equal). **LONGTAO HUANGFU:** Methodology (equal); project administration (equal); writing – original draft (equal); writing – review and editing (equal). **Biao Fan:** Data curation (equal); investigation (equal); writing – original draft (equal). **qianzheng zhuang:** Investigation (equal); methodology (equal); writing – original draft (equal); writing – review and editing (equal). **Liu He:** Investigation (equal); validation (equal); visualization (equal); writing – original draft (equal); writing – review and editing (equal). **Lin Li:** Investigation (equal); methodology (equal). **Wei You:** Investigation (equal); methodology (equal); writing – original draft (equal); writing – review and editing (equal). **Xiaofang Xing:** Investigation (equal); project administration (equal); writing – review and editing (equal).

## FUNDING INFORMATION

This work was supported by National Nature Science Foundation of China (No. 82203579, No.81402308, No. 82272889, and No. 81902985); Natural Science Foundation of Beijing, China (No. 7214215, 7222023); Key Project of National Natural Science Fund Joint Funds (No. U20A20371); Science Foundation of Peking University Cancer Hospital (XKFZ2308, JC202304, 2021‐24); CSCO (Y‐MSDZD2021‐0181); Clinical Medicine Plus X‐Young Scholars Project (PKU2020LCXQ001); Post‐doctoral Research Grant to stay (come) in Shenzhen (1040012), Shenzhen Second People's Hospital 2022 hospital‐level clinical research project (20223357010).

## CONFLICT OF INTEREST STATEMENT

The authors declare that they have no competing interests.

## ETHICS STATEMENT

This study was conducted in accordance with the Declaration of Helsinki and had been authorized by the Ethics Committee of Peking University Cancer Hospital (permission number 2019KT111). Written informed consents from voluntary subjects were obtained.

## Supporting information


Figure S1–S2

## Data Availability

The data that support the findings of this study are available from Peking University Cancer Hospital but restrictions apply to the availability of these data, which were used under license for the current study, and so are not publicly available. Data are however available from the authors upon reasonable request and with permission of Peking University Cancer Hospital.
