# Supplementary material for: T‐cells infiltration mediates the association between neutrophil/lymphocyte ratio and survival in gastric cancer
Source: Cancer Med. 2023 Jun 12;12(15):15893–902. doi: 10.1002/cam4.6228 (PMC10469634; doi:10.1002/cam4.6228)
Supplement: Supplementary file 1 — Figure S1–S2 [file CAM4-12-15893-s001.docx]

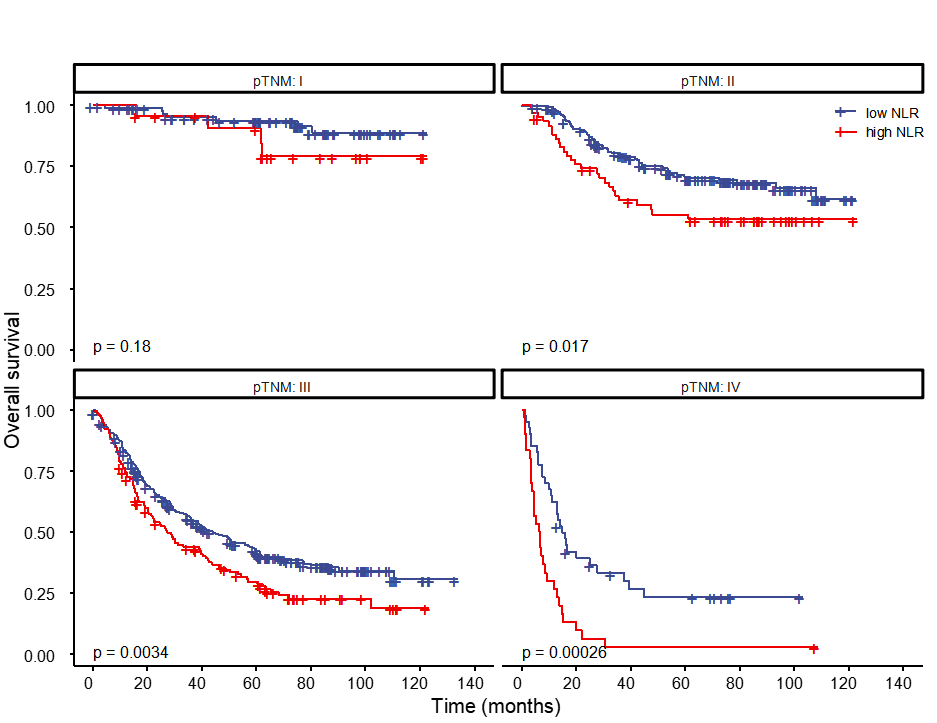


Supplementary Figure 1. Kaplan-Meier curves stratified by pTNM staging, NLR was a prognostic predictor independent from TNM staging system.


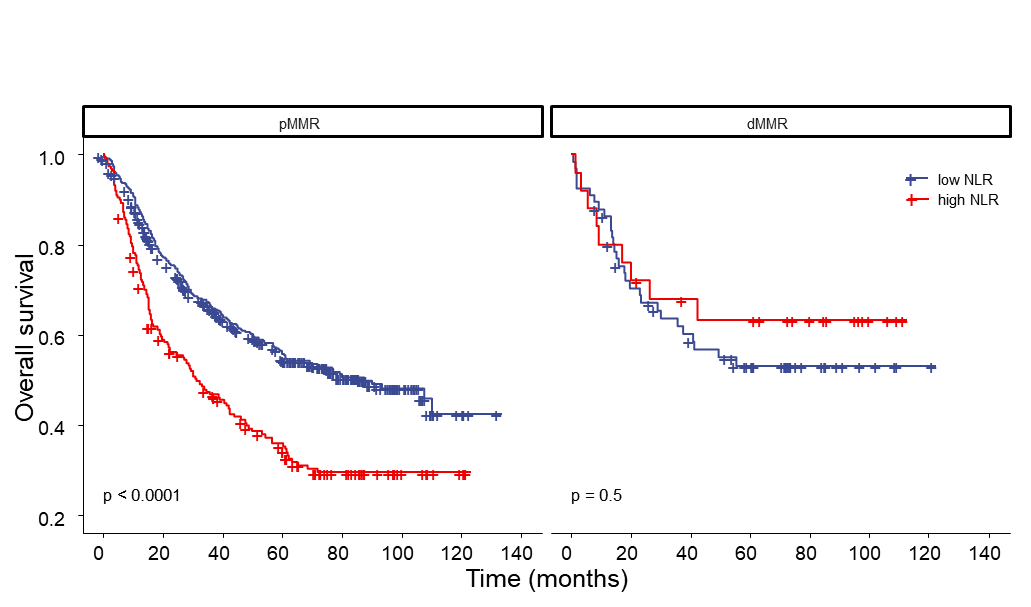


Supplementary Figure 2. Kaplan-Meier curves stratified by DNA mismatch repair (MMR) status. pMMR, proficient mismatch repair; dMMR, deficient mismatch repair.
